# Comparative Transcriptome Analysis of Cultivated and Wild Watermelon during Fruit Development

**DOI:** 10.1371/journal.pone.0130267

**Published:** 2015-06-16

**Authors:** Shaogui Guo, Honghe Sun, Haiying Zhang, Jingan Liu, Yi Ren, Guoyi Gong, Chen Jiao, Yi Zheng, Wencai Yang, Zhangjun Fei, Yong Xu

**Affiliations:** 1 Beijing Key Laboratory of Growth and Developmental Regulation for Protected Vegetable Crops, Department of Vegetable Science, College of Agronomy and Biotechnology, China Agricultural University, Beijing, China; 2 Key Laboratory of Biology and Genetic Improvement of Horticultural Crops (North China), National Engineering Research Center for Vegetables, Beijing, China; 3 Boyce Thompson Institute for Plant Research, Cornell University, Ithaca, NY, United States of America; 4 USDA Robert W. Holley Center for Agriculture and Health, Ithaca, NY, United States of America; Zhejiang University, CHINA

## Abstract

Watermelon [*Citrullus lanatus* (Thunb.) Matsum. & Nakai] is an important vegetable crop world-wide. Watermelon fruit quality is a complex trait determined by various factors such as sugar content, flesh color and flesh texture. Fruit quality and developmental process of cultivated and wild watermelon are highly different. To systematically understand the molecular basis of these differences, we compared transcriptome profiles of fruit tissues of cultivated watermelon 97103 and wild watermelon PI296341-FR. We identified 2,452, 826 and 322 differentially expressed genes in cultivated flesh, cultivated mesocarp and wild flesh, respectively, during fruit development. Gene ontology enrichment analysis of these genes indicated that biological processes and metabolic pathways related to fruit quality such as sweetness and flavor were significantly changed only in the flesh of 97103 during fruit development, while those related to abiotic stress response were changed mainly in the flesh of PI296341-FR. Our comparative transcriptome profiling analysis identified critical genes potentially involved in controlling fruit quality traits including α-galactosidase, invertase, UDP-galactose/glucose pyrophosphorylase and sugar transporter genes involved in the determination of fruit sugar content, phytoene synthase, β-carotene hydroxylase, 9-*cis*-epoxycarotenoid dioxygenase and carotenoid cleavage dioxygenase genes involved in carotenoid metabolism, and 4-coumarate:coenzyme A ligase, cellulose synthase, pectinesterase, pectinesterase inhibitor, polygalacturonase inhibitor and α-mannosidase genes involved in the regulation of flesh texture. In addition, we found that genes in the ethylene biosynthesis and signaling pathway including ACC oxidase, ethylene receptor and ethylene responsive factor showed highly ripening-associated expression patterns, indicating a possible role of ethylene in fruit development and ripening of watermelon, a non-climacteric fruit. Our analysis provides novel insights into watermelon fruit quality and ripening biology. Furthermore, the comparative expression profile data we developed provides a valuable resource to accelerate functional studies in watermelon and facilitate watermelon crop improvement.

## Introduction

Watermelon [*Citrullus lanatus* (Thunb.) Matsum. & Nakai] is an important vegetable crop in the Cucurbitaceae family with sweet and juicy fruit containing high content of lycopene [1]. The production of watermelon accounts for approximately 9.5% of total vegetable production in the world [2]. Watermelon fruit contains a variety of nutrients including fiber, vitamins, antioxidants and minerals, which are essential for human health. The commercial quality of watermelon fruits is determined by many factors such as fruit size and shape, rind color and thickness, flesh color and texture, sugar content, aroma, flavor and nutrient composition [3]. The sweet, colored and juicy fruit makes it the model system for the study of sugar and carotenoid metabolism of non-climacteric fleshy fruit [4].

During the development process, the fruits of cultivated and wild watermelon undergo highly different biochemical and physiological changes such as sugar and pigment accumulation, fruit softening, and changes of flavor and aromatic volatile contents [1, 5], all of which are caused by developmentally and physiologically changes in gene expression profiles. These differences provide an ingenious system to discover molecular mechanisms and candidate genes governing the process of fruit quality development. More importantly, gene expression profiles during fruit development in wild watermelon have not been investigated.

High throughput and low cost of the next-generation sequencing (NGS) technologies offer unique opportunities for genomics and functional genomics research of economically important crops. We have completed the whole genome sequencing of the cultivated watermelon inbred line 97103 [6], which provides an essential basis for downstream functional genomics studies to understand regulatory networks of key biological processes in watermelon.

In this study, we selected cultivated watermelon inbred line 97103 and wild germplasm PI296341-FR for comparative fruit transcriptome analysis. The line 97103 (*C*. *lanatus* subsp. *vulgaris*) is a typical early maturing East Asian cultivar that produces medium size, round shape, thin rind, and green striped fruit with sweet, light red and crispy flesh, which matures at approximately 30 days after pollination (DAP). PI296341-FR (*C*. *lanatus* subsp. *lanatus*) is a wild watermelon that produces round shape, medium size, thick and hard rind, and light green striped fruit with non-sweet and white flesh, which matures at ~50 DAP. *C*. *lanatus* subsp. *lanatus* are distributed in Southern Africa, a region generally regarded as the center of watermelon origin. We have compared and analyzed dynamics of sugar accumulation and related enzyme activities during fruit development of these two subspecies [1]. In this study we performed comparative analysis of fruit transcriptome profiles of the cultivated and wild watermelon, coupled with the integrative analysis of comprehensive profiles of interesting metabolites and enzymatic activities during fruit development. Our analysis provided further insights into the genome-wide gene expression profiles during watermelon fruit quality formation and ripening.

## Materials and Methods

### Plant materials

**Fig 1:**
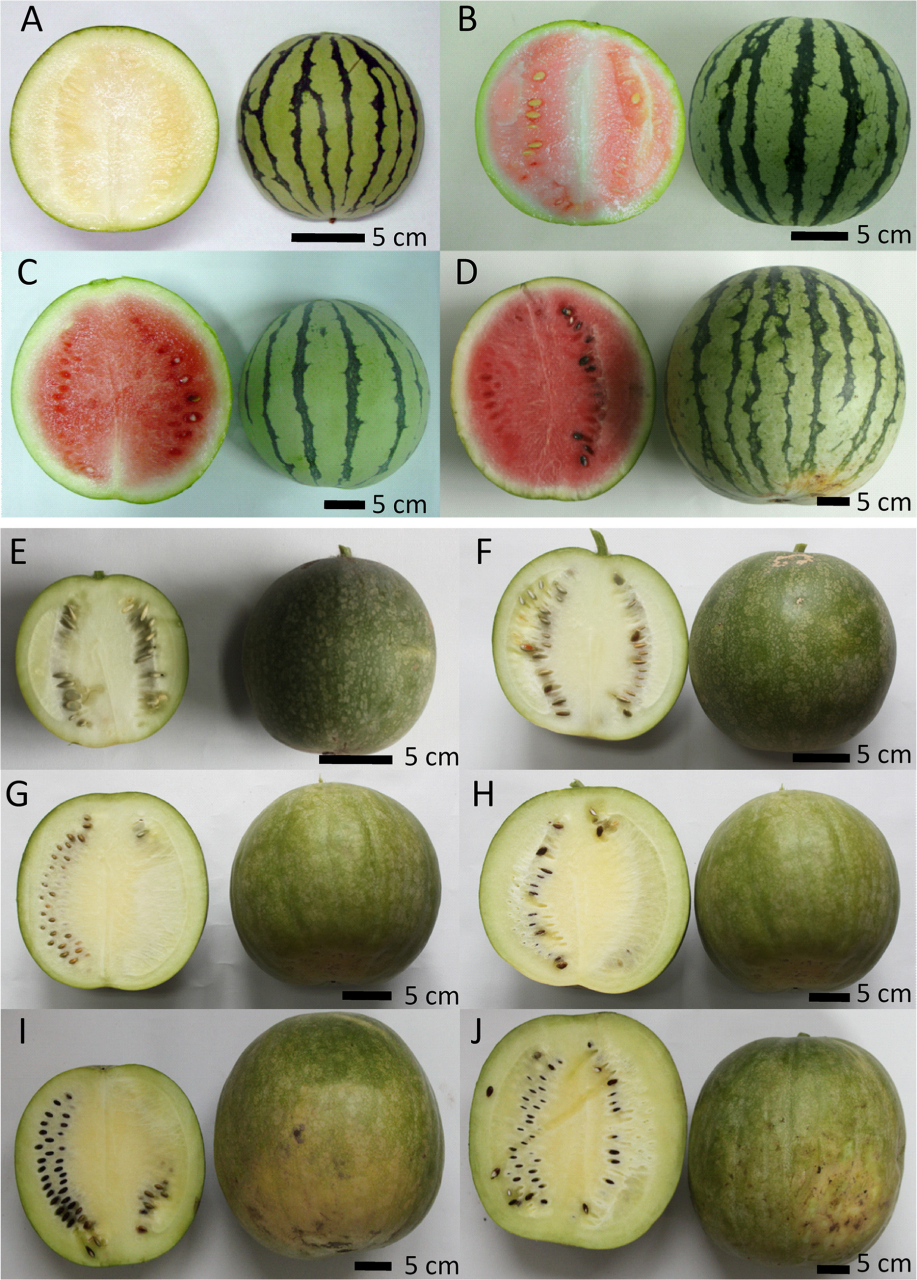
Fruits of the cultivated watermelon 97103 and the wild watermelon PI296341-FR at critical development stages. 97103 fruit: 10 DAP (A), 18 DAP (B), 26 DAP (C) and 34 DAP (D). PI296341-FR fruit: 10 DAP (E), 18 DAP (F), 26 DAP (G), 34 DAP (H), 42 DAP (I) and 50 DAP (J). DAP: days after pollination.

Plants of watermelon *C*. *lanatus* (Thunb.) Matsum. & Nakai subsp. *vulgaris* cv 97103 and *C*. *lanatus* (Thunb.) Matsum. & Nakai subsp. *lanatus* germplasm PI296341-FR were grown in greenhouse in plastic pots containing mixed substrates (peat:sand:pumice, 1:1:1, v/v/v). Flowers were hand-pollinated and tagged. Center flesh and mesocarp samples were collected at 10, 18, 26, 34, 42 and 50 DAP, respectively (Fig 1). Tissues were frozen in liquid nitrogen immediately and stored at -80°C till use.

### Total RNA extraction and RNA-Seq library construction and sequencing

Total RNA was extracted using the Huayueyang Quick RNA isolation Kit (Cat. No.: ZH120; Huayueyang Biotechnology, Beijing, China) following the manufacturer’s instructions. The quantity and quality of the total RNA were checked by a NanoDrop 1000 spectrophotometer (Thermo Fisher Scientific Inc.; USA) and by resolution on a 1% non-denaturing agarose gel, respectively. Strand-specific RNA-Seq libraries were prepared following the protocol described in Zhong et al. [7] and sequenced on an Illumina HiSeq 2000 system using the single-end, 100 bp mode. Two biological replicates were performed for each sample. The raw sequencing data has been deposited in NBCI SRA under the accession numbers SRP012849 and SRP051354.

### Identification of differentially expressed genes

Raw RNA-Seq reads were first processed to eliminate adapter and low quality sequences using Trimmomatic [8]. The reads were then aligned to a ribosome RNA (rRNA) database [9] using Bowtie [10] allowing up to three mismatches and those aligned to rRNA sequences were discarded. The resulting high-quality cleaned reads were aligned to watermelon genome sequences [6] using Tophat allowing one segment mismatch [11]. Following alignments, the number of reads mapped to each watermelon gene model was derived, and then normalized to reads per kilobase of exon model per million mapped reads (RPKM). To identify differentially expressed genes (DEGs) during the fruit development, the raw counts were first transformed using the getVarianceStabilizedData module in DESeq [12]. Then the transformed expression data were fed to LIMMA [13], and F tests were performed. Raw P values were adjusted for multiple testing using the Benjamini-Hochberg procedure [14]. GO term enrichment analysis of DEGs was performed using GO::TermFinder [15], with adjusted p values being less than 0.01. Significantly changed pathways were identified using the Plant MetGenMAP system [16].

### qRT-PCR analysis

qRT-PCR assays were performed with the LightCycler480 RT-PCR system (Roche, Switzerland) to validate the gene expression determined by RNA-Seq analysis. Each reaction consisted of 10 μL SYBR Green I Master Mix, 5 μL cDNA (20 ng/uL) and 5 μL primer mix (2 μM of each primer). Reactions were performed at 95°C for 3 min, followed by 45 cycles of 95°C for 10 sec, 58°C for 20 sec, and 72° C for 30 sec. Melting curves were performed to detect primer dimers. The 18S rRNA gene was used as the internal control. Quantification was performed by normalizing the number of target genes to 18S rRNA gene using the comparative Ct method [17]. The ΔCt was calculated by subtracting the average Ct of each tissue type from the average Ct of 18S rRNA. The ΔΔCt was calculated by subtracting the ΔCt of each fruit stage from the ΔCt of the fruit tissue. The formula 2^-(ΔΔCt) was used to calculate the relative fold change between the fruit development stages. Primer pairs used for qRT-PCR analysis are listed in S1 Table.

### Determination of the content of carotenoids

The contents of lycopene, β-carotene and lutein were determined following the protocol of Fraser et al. [18]. Individual carotenoids were separated by HPLC using Waters Nova-Pak C18 column.

### Determination of the soluble solid content and firmness of mesocarp

Soluble solid content of mesocarp tissues in both 97103 and PI296341-FR was determined using a hand-held refractometer ATC-1E refractometer (ATAGO, Tokyo) following the manufacturer’s instructions. The mesocarp firmness was determined using the FT-327 penetrometer (Bertuzzi, Facchini, Italy).

## Results and Discussion

### Transcriptome sequencing

We previously generated high-throughput RNA-Seq data from the flesh and mesocarp of 97103 fruit at four critical fruit development stages (10 DAP, 18 DAP, 26 DAP and 34 DAP) [6]. In the present study RNA-Seq data was also generated from the flesh of PI296341-FR fruit, to explore the differences of transcriptome dynamics during fruit development between 97103 and PI296341-FR. The mesocarp of PI296341-FR fruit was not included in this study since there is no significant difference regarding fruit quality related phenotypes such as sugar content, color and texture, between the mesocarp of PI296341-FR and 97103 (Fig 1 and S2 Table). Four critical fruit development stages, immature white (10 DAP), white-pink flesh (18 DAP), red flesh (26 DAP) and full-ripe (34 DAP), were examined for 97103. Two additional stages, 42 DAP and 50 DAP, of PI296341-FR were examined considering its late fruit maturity (Fig 1).

The high-throughput Illumina strand-specific RNA sequencing technology was employed to sequence the 14 samples (two biological replicates for each sample). After trimming adaptors and removing low quality sequences and rRNA contaminated reads, a total of 321.8 million high quality, cleaned reads were obtained, with each library having at least 8 million reads. This high-quality RNA-Seq dataset provided a solid foundation for our comparative transcriptome analysis in an attempt to identify key genes involved in regulating watermelon fruit quality and ripening process.

### Identification of differentially expressed genes during cultivated and wild watermelon fruit development

**Fig 2:**
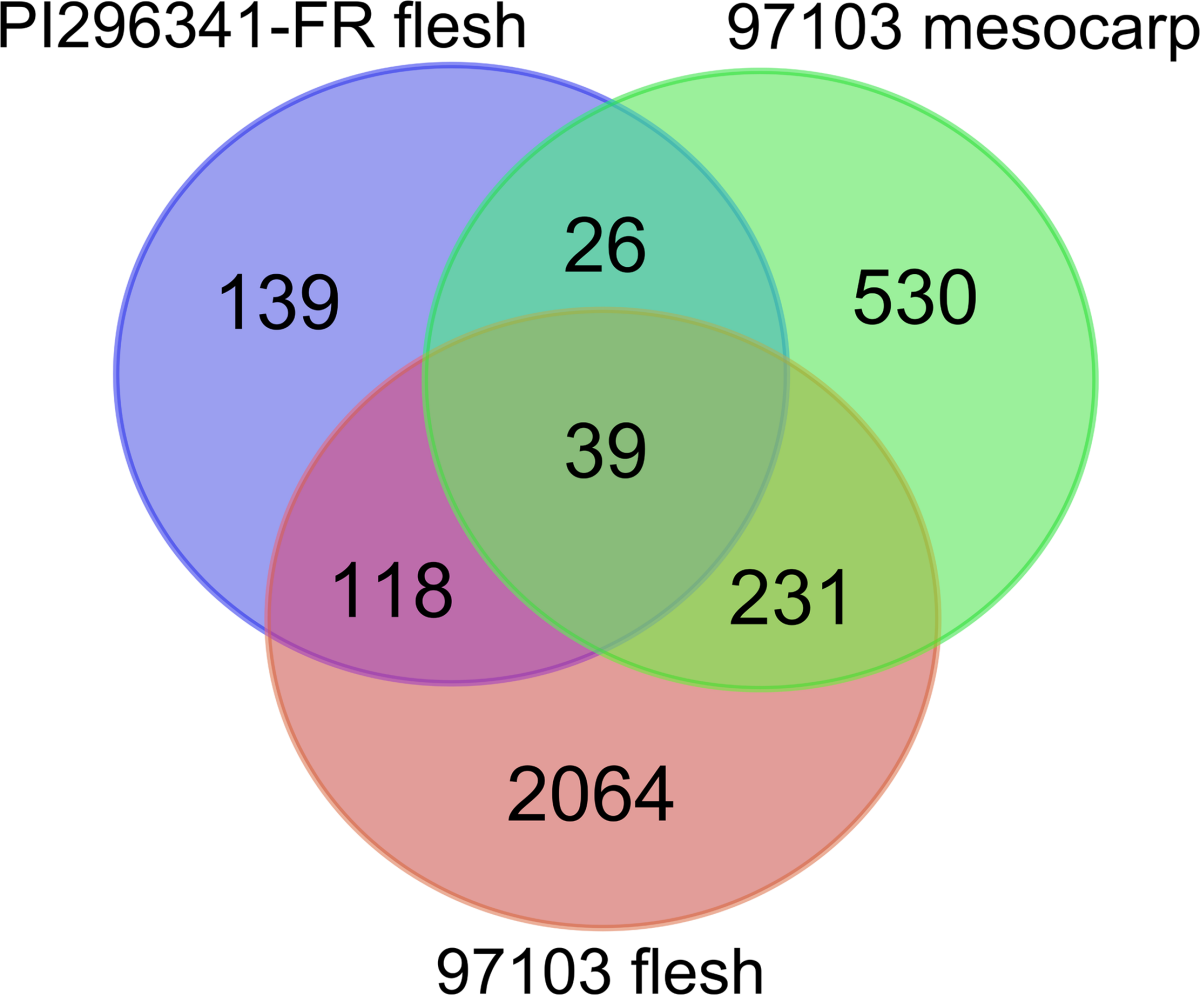
Venn diagram of differentially expressed genes in three tissues during watermelon fruit development.

RNA-Seq is powerful and efficient for large-scale gene expression analysis. In this study, we found that 18,198 (77.6%), 18,392 (78.5%) and 17,954 (76.6%) genes were expressed in the flesh and mesocarp of 97103 fruit and the flesh of PI296341-FR fruit, respectively. The number of genes covered by our RNA-Seq data was comparable to that in other RNA-Seq studies [19]. Gene expression analysis identified 2,452, 826 and 322 genes that were differentially expressed in the flesh and mesocarp of 97103 and flesh of PI296341-FR, respectively, during fruit development and ripening (S3 Table). Further analysis indicated that 2,064, 530 and 139 genes were specifically differentially expressed in the flesh and mesocarp of 97103 fruit and the flesh of PI296341-FR fruit, respectively (Fig 2 and S3 Table). As expected, many more genes were differentially expressed in the flesh of 97103, in concordance with the fact that a series of physiological and biochemical changes, such as those in color, texture and sugar content, were only observed in the flesh of 97103 during fruit development. These results reflected more complicated regulatory networks of gene expression in the 97103 fruit flesh.

To validate gene expression levels determined by RNA-Seq analysis, qRT-PCR assays were performed on six DEGs involved in sugar metabolism, pigment accumulation and endogenous ethylene biosynthesis during fruit development in cultivated and wild watermelon. The results showed that the gene expression trends of selected genes detected by RNA-Seq and qRT-PCR analyses were largely consistent (S4 Table). Actually, the high accuracy of RNA-Seq in determining gene expression levels and the comparability between results of RNA-Seq and qRT-PCR have been widely validated [19–22]. These results further confirmed the robustness of our gene expression data.

### Functional analysis of differentially expressed genes

To gain further insights into the molecular mechanisms of attractive quality formation during watermelon fruit development, gene ontology (GO) enrichment analysis was performed on DEGs to characterize the differences of fruit development and ripening between the cultivated and wild watermelon. We found that genes related to biological processes such as cellular amino acid derivative metabolic process, cellular metabolic process and phenylpropanoid metabolic process were highly enriched in DEGs from at least two of the three investigated tissues. These genes are involved in the basic substance and energy metabolism and response actions during watermelon fruit development (S5 Table). More importantly, genes related to biological processes involved in watermelon fruit quality development and ripening were only enriched in the flesh of 97103 fruit, such as aromatic compound biosynthetic process, carbohydrate metabolic process, fatty acid metabolic process, hexose metabolic process, lipid metabolic process, monosaccharide metabolic process, organic acid metabolic process, and secondary metabolic process. Differential expression of these genes in the flesh of 97103 should be crucial for fruit quality differences between the cultivated and wild watermelon. Very few biological processes were found to be enriched in DEGs of PI296341-FR flesh development. These biological processes were mainly related to abiotic stress responses such as response to oxidative stress, high light intensity, heat acclimation, reactive oxygen species, ethanol, arsenic, cadmium ion, and hydrogen peroxide (S5 Table), consistent with the fact that PI296341-FR is more tolerant to various environmental stresses.

### Significantly changed biochemical pathways

Fruit development is a genetically programmed event defined by a series of physiological and biochemical changes that ultimately alter fruit color, texture, and aroma and nutrition components. In total, 204, 88 and 34 pathways were represented by DEGs in the flesh and mesocarp of 97103 and the flesh of PI296341-FR, respectively, among which 50, 17 and 5 were significantly changed (S6 Table). Several fruit quality or signal transduction related pathways, such as cellulose biosynthesis, UDP-D-xylose biosynthesis, melibiose degradation, sucrose degradation, brassinosteroid biosynthesis, phenylalanine biosynthesis, flavonoid biosynthesis, plant sterol biosynthesis and spermine biosynthesis, were significantly changed only in the flesh of cultivated watermelon 97103. These significantly changed pathways in the cultivated watermelon flesh should be the key metabolic networks leading to the fruit quality difference between the cultivated and wild watermelon. Only two unique pathways, glutamine biosynthesis and nitrate reduction VI (assimilatory), were significantly changed in the flesh of PI296341-FR. These pathways may contribute to the higher vigor of the wild watermelon than that of the cultivated watermelon [23, 24].

### Comparison of gene expression profiles during fruit development and ripening

The maturation and ripening of watermelon fruit are highly coordinated developmental programs. One of the key phases of fruit development is ripening, a slowed and prolonged form of senescence. When fruits matured physiologically, their growth terminates and the ripening process is initiated. The ripening process leads to fruit cellular metabolism changes, causes the development of an edible soft fruit with attractive qualities.

#### Sugar metabolism and accumulation

The sweetness of cultivated watermelon fruit flesh is among its most attractive characteristics. Sugars are the main component of the dry matter of watermelon fruit flesh and are highly accumulated in the fruit flesh of cultivated watermelon 97103 during ripening. In contrast, the wild PI296341-FR has no obvious changes in fruit flesh sweetness [1]. Sugar content in watermelon fruit is determined by phloem unloading and metabolism within the fruit flesh. Stachyose, raffinose and sucrose are the main sugars transported in the phloem of cucurbit plants [25, 26]. Stachyose and raffinose are the main sugars transported to the fruit sink, where they are rapidly metabolized [27]. A total of 62 sugar metabolism genes were annotated in the watermelon genome. Twenty-two genes, including those encoding α-galactosidase, invertase and UDP-galactose/glucose pyrophosphorylase (UDP-Gal/Glc PPase) were differentially expressed during fruit development and ripening (S7 Table).

**Fig 3:**
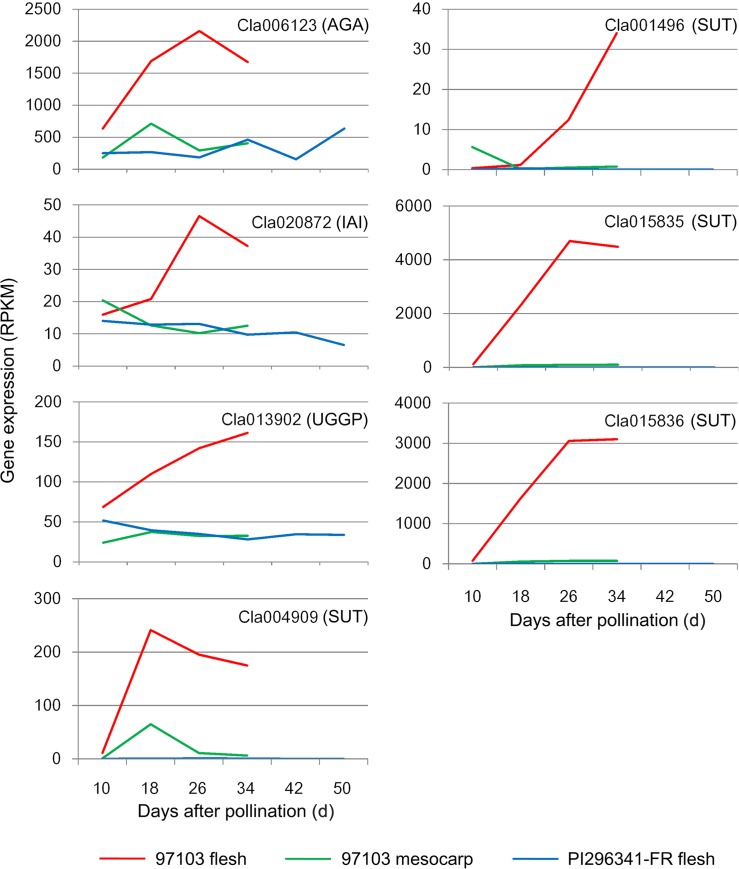
Expression profiles of sugar metabolism genes during watermelon fruit development. AGA: α-galactosidase; IAI: insoluble acid invertase; UGGP: UDP-Gal/Glc PPase; SUT: sugar transporter.

α-galactosidase is the main enzyme hydrolyzing stachyose and raffinose and determining sink strength in cucurbit plants [28]. A total of nine α-galactosidase genes were identified in the watermelon genome [6]. We found that the expression of one of them, *Cla006123*, in 97103 flesh was 3–9 fold higher than that in PI296341-FR flesh and 97103 mesocarp during fruit development and ripening (Fig 3). *Cla006123* showed high identity (94%) at the amino acid sequence level to the melon alkaline α-galactosidase (S1 Fig), *CmAGA2*, which functions in key processes of galactosyl-oligosaccharide metabolism, such as raffinose family oligosaccharide (RFO) photosynthate translocation [29]. These results indicated that the *Cla006123* gene might be a key element involved in phloem unloading and sink strength determination during watermelon fruit development.

In sucrose-translocating plants, such as tomato and carrot [30, 31], both phloem unloading and sucrose translocation to fruit sinks need insoluble acid invertase. Our previous study indicated that the enzyme activity of insoluble acid invertase and sucrose content were highly correlated in the fruit flesh tissue of 97103 and PI296341-FR [1]. A total of five insoluble acid invertase genes were found in the watermelon genome. One of them, *Cla020872*, was up-regulated in the 97103 flesh before the full-ripe stage. In contrast, its expression remained constant and was much lower in 97103 mesocarp and PI296341-FR fruit flesh (Fig 3). The expression profile of *Cla020872* was positively correlated with the enzyme activity of insoluble acid invertase in the flesh and the mesocarp of 97103 and the flesh of PI296341-FR [1]. These results supported that *Cla020872* could be involved in the extracellular sucrose degeneration which facilitates fructose and glucose translocation and the intercellular sugar accumulation in watermelon fruit.

As the product of RFO catabolism, galactose is the key regulatory element of sugar metabolism in cucurbits [32]. In the proposed pathway of galactose metabolism, UDP-Gal/Glc PPase plays an important role in melon fruit sink metabolism by catalyzing UDP-Gal synthesis from Gal-1-P and the subsequent reaction from UDP-Glc to Glc-1-P [32]. A UDP-Gal/Glc PPase gene, *Cla013902*, was consistently up-regulated in the flesh of 97103 during watermelon fruit development, while its expression kept relatively constant and was lower in the flesh of PI296341-FR and 97103 mesocarp, indicating that *Cla013902* may contribute to fruit sugar metabolism in watermelon (Fig 3).

A total of 23 sugar transporter genes differentially expressed during fruit development and ripening in 97103 and PI296341-FR were identified in the watermelon genome (S7 Table). Two *SWEET* like sugar transporters (*Cla004909* and *Cla001496*) and two major facilitator superfamily sugar transporters (*Cla015835* and *Cla015836*) were highly up-regulated in the flesh of 97103. In contrast, their expression in 97103 mesocarp was much lower than that in 97103 flesh and almost undetectable in the PI296341-FR flesh tissue (Fig 3). In addition, their expression profiles were highly correlated with sugar accumulation in watermelon fruit [1]. Moreover, *Cla015835* and *Cla015836* were located at the flanking region of the fruit sugar content major QTL *Qbrix2-2* on watermelon chromosome 2 [33]. These results suggested that these transporter genes might play an important role in increasing the fruit flesh sweetness by facilitating the active transmembrane transport of sugars.

In summary, our comprehensive comparative analysis of the fruit transcriptome dynamics between the cultivated and wild watermelon provided novel clues to help us understand the complex gene networks involved in sugar unloading, metabolism and partitioning during sugar accumulation in watermelon fruit.

#### Flesh carotenoid biosynthesis and metabolism

Recently increasing studies focus on the carotenoid regulatory networks of food crops considering their nutrition value for human health [34]. Watermelon is one of the few species that accumulate a large amount of carotenoids in its fruits. The major carotenoid accumulated in red flesh watermelon is lycopene and its average concentration is approximately 60% more than that in tomato fruit [35]. In this study, we determined the content of lycopene, β-carotene and lutein in the fruit flesh of the cultivated watermelon 97103 and the wild watermelon PI296341-FR. The lycopene and β-carotene content in the 97103 flesh progressively increased during fruit development and ripening, whereas their content in the flesh of PI296341-FR remained relatively constant and was much lower at the later stages of fruit development. The content of lutein in 97103 flesh increased slightly while displayed no significant difference between 97103 and PI296341-FR (S8 Table).

**Fig 4:**
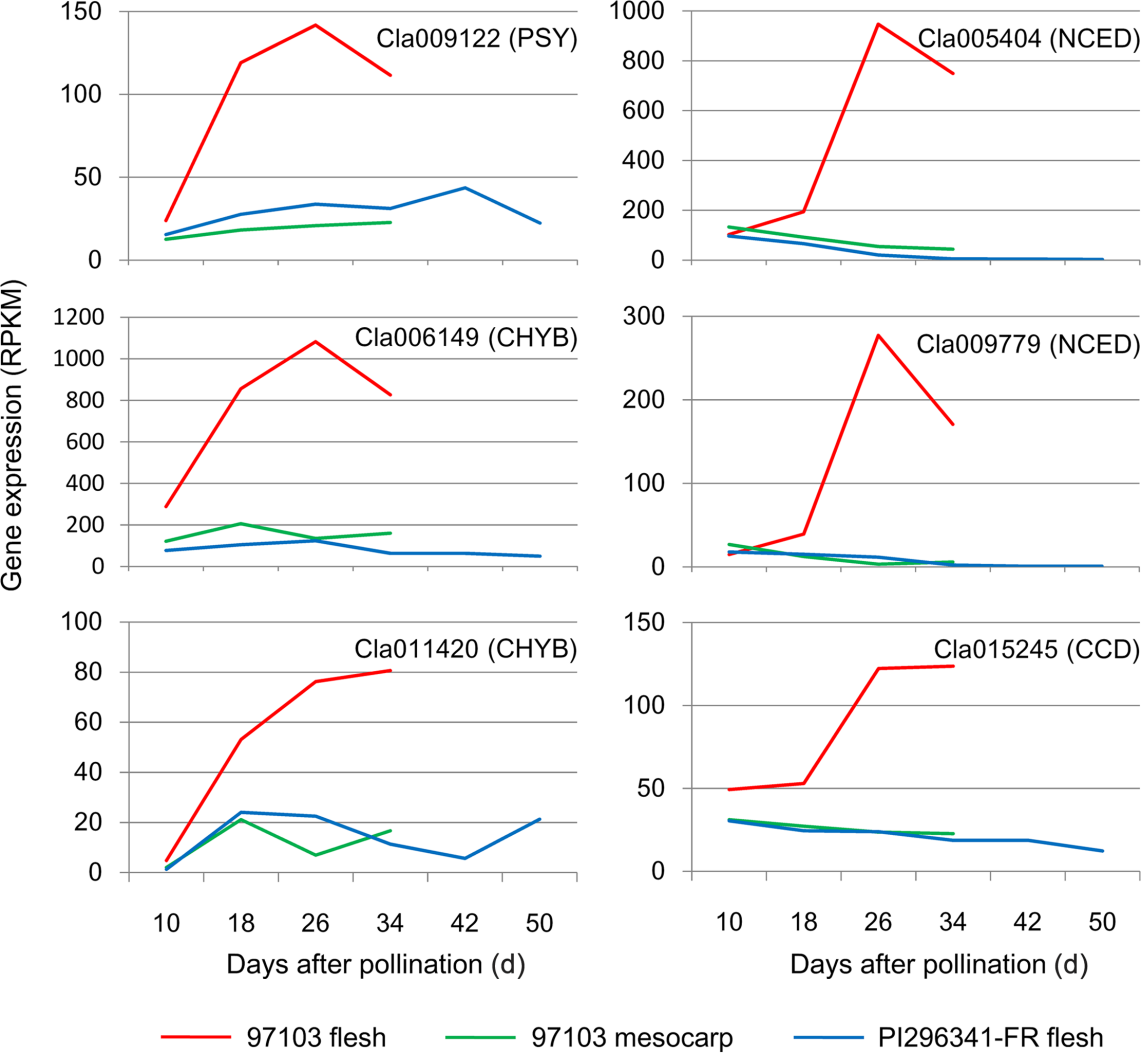
Expression profiles of carotenoid biosynthesis and metabolism genes during watermelon fruit development. PSY: phytoene synthase; CHYB: β-carotene hydroxylase; NCED: 9-*cis*-epoxy-carotenoid dioxygenase; CCD: carotenoid cleavage dioxygenase.

A total of 26 carotenoid metabolism pathway genes were identified in the watermelon genome, among which seven were differentially expressed during fruit ripening, including phytoene synthase, β-carotene hydroxylase, 9-*cis*-epoxycarotenoid dioxygenase and carotenoid cleavage dioxygenase genes (S9 Table). There are three phytoene synthase genes in the watermelon genome. The expression of one of them, *Cla009122*, in fruit flesh of 97103 was very low at 10 DAP (white flesh) but increased dramatically and peaked at 26 DAP (red flesh), and then decreased slightly at 34 DAP. This is consistent with the finding in the watermelon cultivar Dumara [19]. In contrast, the expression of *Cla009122* in the flesh of PI296341-FR and mesocarp of 97103 was stable during fruit development and much lower than that in 97103 flesh (Fig 4). Our results confirmed that *Cla009122*, which corresponds to the tomato *PSY-1* gene, plays an important role in determining watermelon flesh color.

The watermelon genome contains three β-carotene hydroxylase genes. Two of them, *Cla006149* and *Cla011420*, were differentially expressed during watermelon fruit development. They were highly up-regulated in the flesh of 97103 during fruit development and ripening. Similar trends were also found in the watermelon cultivar Dumara [19]. In contrast, the expression of the two β-carotene hydroxylase genes in the flesh of PI296341-FR and mesocarp of 97103 was stable and significantly lower than that in 97103 flesh in fruit ripening process (Fig 4). This result suggested that the higher expression level of *Cla006149* and *Cla011420* in 97103 might help to maintain the sustainable concentration of carotenes as intermediate metabolites for the generation of downstream compounds.

9-*cis*-epoxy-carotenoid dioxygenase (NCED) is involved in the catabolic process which converts 9-*cis*-violaxanthin or 9-*cis*-neoxanthin to xanthoxin, a precursor of plant hormone abscisic acid (ABA) [36, 37]. Four NCED genes were identified in the watermelon genome. The expression of two of them, *Cla005404* and *Cla009779*, in the flesh of 97103 was significantly up-regulated during fruit development and peaked at 26 DAP; in contrast, their expression decreased in the flesh of PI296341-FR and 97103 mesocarp during fruit development and was much lower than that in the corresponding 97103 flesh tissues (Fig 4). The up-regulated expression of the two NCED genes may facilitate the streaming from carotenoid metabolism to ABA metabolism during fruit development, consistent with the increased ABA content in watermelon fruit during its development and ripening as reported previously [38].

Carotenoid cleavage dioxygenase (CCD) catalyzes the oxidative cleavage of carotenoids and leads to the production of apocarotenoids. This process helps to maintain the stable level of carotenoids in tissues [39]. Apocarotenoids, including β-cyclocitral, β-ionone, geranial, geranyl acetone, theaspirone, α-damascenone and β-damascenone, all contribute to fruit flavor. There are three CCD genes in the watermelon genome. We found that one of them, *Cla015245*, was highly up-regulated during fruit development in the flesh of 97103 and its expression peaked at 26 DAP and 34 DAP, the best fruit quality stages, while its expression kept constantly lower in the flesh of PI296341-FR and 97103 mesocarp (Fig 4). These results indicated that Cla015245 might function as an important tie between the flesh pigmentation and flavor during watermelon fruit development and ripening.

Our genome wide comparative expression analysis of the carotenoid biosynthesis and metabolism pathway genes suggests the complex gene expression and regulatory networks generating a flux of lycopene accumulation and consumption during watermelon fruit development and ripening process.

#### Flesh texture

Fruit flesh texture, such as firmness and crispness, is an important quality trait because it is directly related to fruit commercial values including mouth feel, fruit storability, transportability and shelf-life. Fruit softening and textural changes during ripening are mainly caused by progressive cell wall depolymerization and solubilization and loss of cell structure. Fruit cell walls are interlaced networks consisting of polysaccharides and proteins. The fruit primary cell wall contains approximately 35% pectin, 25% cellulose, 20% hemicellulose, and 10% structural protein [40]. Fruit softening, caused by cell wall degradation and intercellular adhesion reduction, is the precondition to forming the attractive high-quality fruits during fruit development and ripening.

Cell wall modifications include de-esterification and depolymerization, and consequently loss of galacturonic acid and neutral sugars followed by solubilization of oligosaccharides and remaining sugar residues [41–43]. These changes lead to the progressive degradation of cell wall polymers and the loss of integrity of the middle lamella, which is rich in pectins that control cell-to-cell adhesion, thus influencing fruit texture [44].

We previously determined the firmness, the content of pectin and crude fiber and the activity of the fruit softening related enzymes in the flesh of 97103 and PI296341-FR [45]. Our transcriptome analysis here identified a total of 79 cell wall metabolism genes that were differentially expressed during fruit development, including 4-coumarate:coenzyme A ligase (4CL), cellulose synthase, pectinesterase, pectinesterase inhibitor, polygalacturonase inhibitor and α-mannosidase genes (S10 Table).

**Fig 5:**
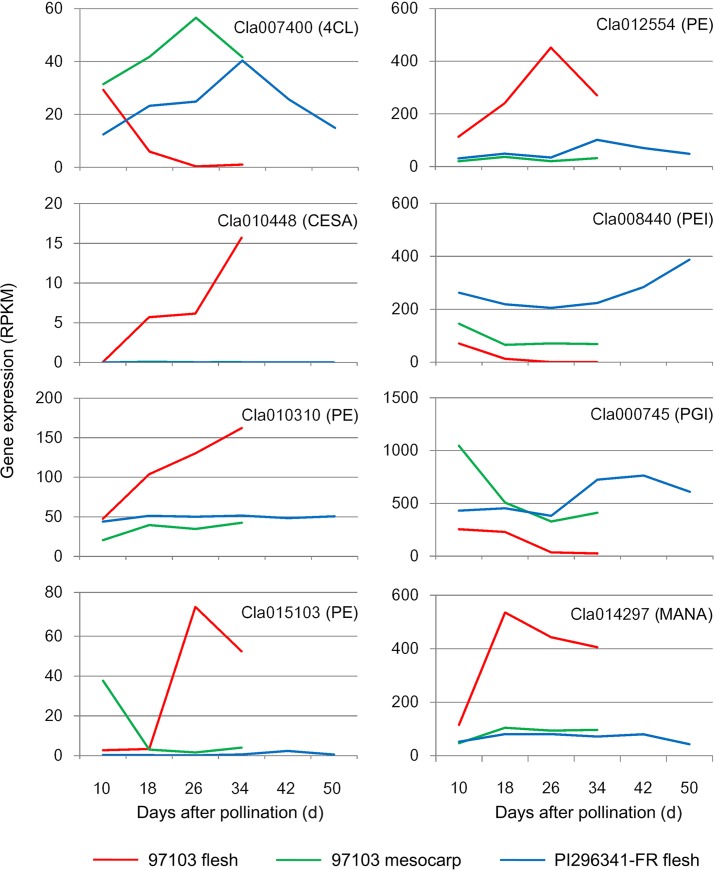
Expression profiles of flesh texture related genes during watermelon fruit development. 4CL: 4-coumarate:coenzyme A ligase; CESA: cellulose synthase; PE: pectinesterase; PEI: pectinesterase inhibitor; PGI: polygalacturonase inhibitor; MANA: α-mannosidase.

Lignification has a negative impact on fruit commercial quality, leading to adverse effects on fruit digestibility. 4CL is a key enzyme in lignin biosynthesis [46]. There are seven 4CL genes in the watermelon genome. One of them, *Cla007400*, showed significantly decreased expression in the 97103 flesh during fruit development; whereas its expression displayed no significant changes during fruit development but was much higher in the flesh of PI296341-FR and mesocarp of 97103 (Fig 5). These results indicated that the 4CL gene *Cla007400* might play an important role in determining the watermelon fruit texture and firmness by regulating the lignin biosynthesis.

Cellulose is the major component of plant cell walls, providing mechanical strength to the structural framework of plants. Cellulose synthase play a key role in the biosynthesis of cellulose [47]. A total of 13 cellulose synthase genes were found in the watermelon genome. One of them, *Cla010448*, showed increased expression in the 97103 flesh during fruit development and ripening while this gene was not expressed or expressed at a very low level in the flesh of PI296341-FR and mesocarp of 97103 (Fig 5). The unique expression pattern of *Cla010448* indicated that this gene might be involved in regulating the construction of cell wall architecture in the 97103 fruit flesh during fruit ripening.

Pectins are the major components of the primary cell wall and middle lamella, determining fruit texture and quality. Pectin depolymerization was reported as the main reason for the loss of fruit firmness [48, 49]. The enzymes mainly responsible for the pectin changes in fruit cell wall are pectinesterase and polygalacturonase. Pectinesterase catalyzes the hydrolytic de-esterification of pectins causing pectic chain esterification, which is further hydrolyzed to pectate by polygalacturonase, causing tissue softening during fruit ripening. Comparative analysis of the fruit transcriptomes identified three watermelon pectinesterase genes, *Cla010310*, *Cla015103* and *Cla012554*, whose expression was increased in the flesh of 97103 during the fruit ripening process and was much higher than that in the flesh of PI296341-FR and mesocarp of 97103 (Fig 5). The activity of pectinesterases can be regulated by pectinesterase inhibitors [50, 51], which bind to the active site of pectinesterases, generating a 1:1 complex [52]. Seven pectinesterase inhibitor genes were identified in the watermelon genome. One gene, *Cla008440*, showed higher expression level in the flesh of PI296341-FR compared to that in the flesh of 97103. In addition, this gene was down-regulated in the flesh of 97103 during fruit ripening (Fig 5). Polygalacturonase is the main enzyme causing pectin solubilization by hydrolyzing α-1,4-glycosidic bonds that hold galacturonic acid residues in de-esterified galacturonan chains produced by pectinesterase. Polygalacturonase inhibitor proteins have been found in the cell wall of dicotyledonous plants. Two polygalacturonase inhibitor genes were found in the watermelon genome. One of them, *Cla000745*, was up-regulated in PI296341-FR flesh whereas its expression in the 97103 flesh was significantly lower than that in PI296341-FR flesh and was decreased throughout the fruit development process (Fig 5). The above results suggested that these genes might play critical roles in regulating the pectic composition of watermelon fruit cell wall during the ripening process.

A total of three α-mannosidase genes were identified in the watermelon genome. One of them, *Cla014297*, had stably low expression throughout the fruit development and ripening in the PI296341-FR flesh and 97103 mesocarp; whereas its expression was up-regulated in the 97103 flesh tissue (Fig 5). It has been reported that acidic α-mannosidase activity increased during fruit ripening in mango and tomato and was highly associated with fruit softening and ripening [53, 54]. The expression pattern of watermelon α-mannosidase gene *Cla014297* suggested that it might be one of the key genes determining fruit texture and firmness.

#### Ethylene biosynthesis and signal transduction

Fruits have been classified as climacteric and nonclimacteric based on the presence or absence of massively increased ethylene synthesis and an accompanying rise in the respiration rate during ripening [55]. Ethylene has been regarded as the ripening hormone in climacteric fruits; however, more and more evidence implies that ethylene is also involved in the ripening of nonclimacteric fruits such as strawberry and citrus [56–58]. Watermelon is a nonclimacteric fruit but its fruit is very sensitive to exogenous ethylene, exhibiting extensive placental and pericarp softening following short exposure to the ethylene gas [59, 60], indicating a possible role of ethylene in regulating watermelon fruit quality.

**Fig 6:**
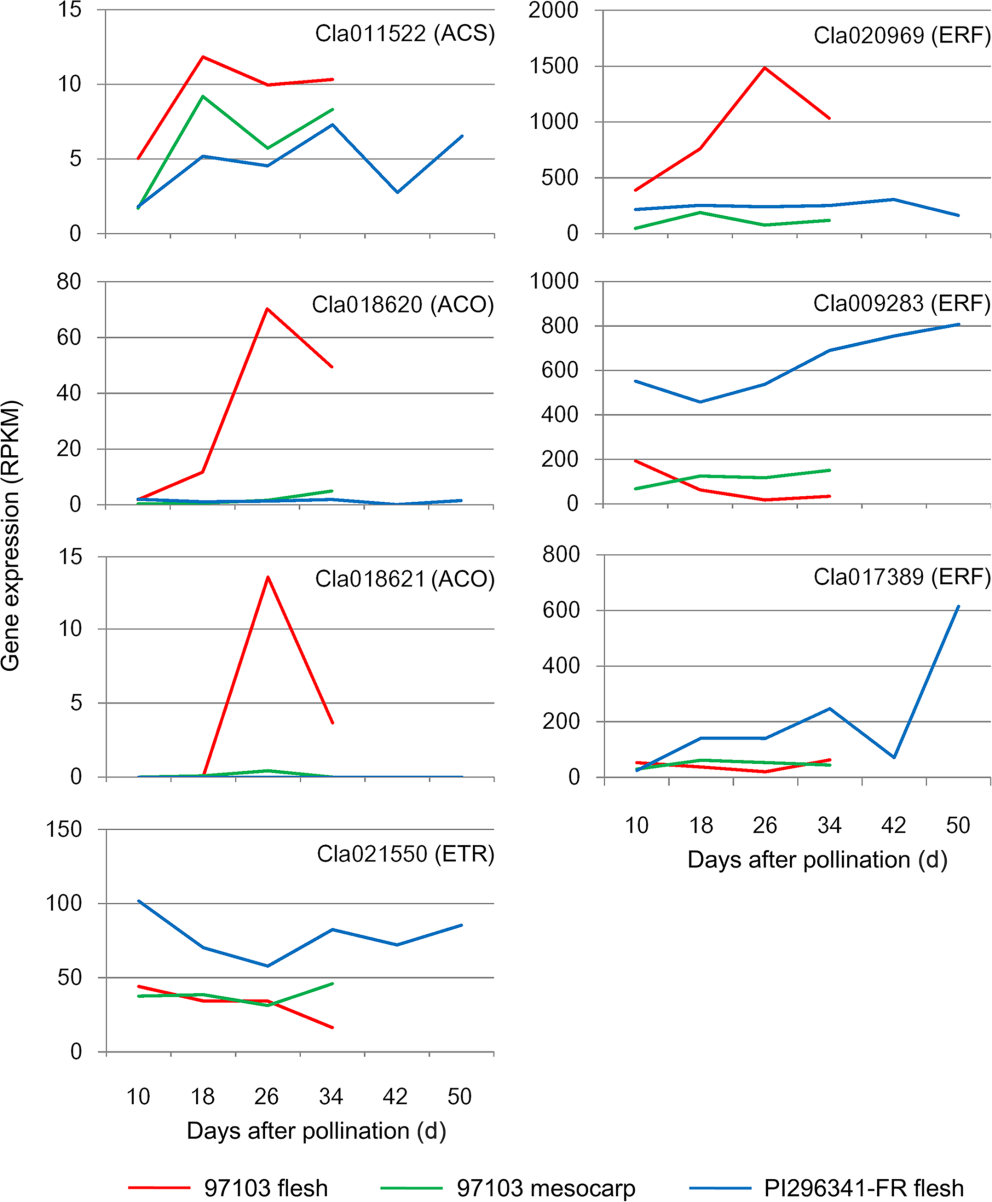
Expression profiles of ethylene biosynthesis and signal transduction genes during watermelon fruit development. ACS: ACC synthase; ACO: ACC oxidase; ETR: ethylene receptor; ERF: ethylene responsive factor.

During the process of fruit development and ripening of 97103 and PI296341-FR, 22 genes in the ethylene biosynthesis and signaling pathway were identified as differentially expressed (S11 Table), among which were ACC synthase (ACS), ACC oxidase (ACO), ethylene receptor (ETR) and ethylene responsive factor (ERF) genes. *ACS* and *ACO* are key genes in the ethylene biosynthesis pathway. ACS has eight homologs in the watermelon genome. One of them, *Cla011522*, was differentially expressed in 97103 mesocarp, while no significant differential expression of *Cla011522* was observed in the flesh of 97103 and PI296341-FR during watermelon fruit development and ripening. However, its expression profiles during fruit development were largely similar in 97103 and PI296341-FR and its expression level in PI296341-FR flesh was relatively lower than that in 97103 flesh (Fig 6). The expression of all other ACS genes (*Cla000483*, *Cla006245*, *Cla006634*, *Cla011230*, *Cla014057*, *Cla014652* and *Cla022653*) was also low in watermelon fruit (Data not shown). It was reported that the concentration of released ethylene in watermelon fruit was significantly lower than that in tomato fruit [3, 61]. Our data suggested that the low level of released ethylene in watermelon fruit might be due to the low expression level of the ACS genes. ACO has five homologs in the watermelon genome. Two ACO genes, *Cla018620* and *Cla018621*, were differentially expressed in 97103 flesh and their expression peaked at 26 DAP; while the expression of these ACO genes was not significantly changed in 97103 mesocarp and PI296341-FR and was much lower than that in 97103 flesh (Fig 6). Differential expression patterns of these ACO genes indicated that they may be involved in the phenotype changes of watermelon fruit by regulating endogenous ethylene biosynthesis during fruit development and ripening. In addition, one of the three ETR homologous genes, *Cla021550*, was down-regulated in 97103 flesh during fruit development and ripening, and its expression level in PI296341-FR flesh remained constantly higher (Fig 6). ETR act as a negative regulator of ethylene responses in tomato [62]. The relatively high expression of *Cla021550* in PI296341-FR flesh may decrease its sensitivity to ethylene.

ERFs belong to the AP2/ERF superfamily, one of the largest transcription factor families in plants, and represent the last step in the ethylene signaling pathway. More and more studies have shown the possible involvement of ERF proteins in fruit ripening. In tomato, an ERF gene, *SlERF6*, has been shown to play an important role in controlling carotenoid accumulation and fruit ripening [22]. In banana, two ERFs, *MaERF9* and *MaERF11*, were suggested to be involved in fruit ripening via transcriptional regulation of or interaction with ethylene biosynthesis genes [63]. In the present study, we identified a total of 16 ERF genes that were differentially expressed during fruit development and ripening and these ERFs genes showed distinct expression patterns in the cultivated and wild watermelon (S11 Table). An ERF gene, *Cla020969*, was highly expressed and its expression was increased during fruit ripening in 97103 flesh; whereas its expression was relatively lower and stable in PI296341-FR flesh and 97103 mesocarp. Two additional ERF genes, *Cla009283* and *Cla017389*, were highly expressed in PI296341-FR, while down-regulated during the ripening in 97103 flesh (Fig 6).

In summary, the highly ripening-associated expression patterns of genes in the ethylene biosynthesis and signaling pathway further indicated a possible role of ethylene in watermelon fruit development and ripening. However, due to the fact that ACS genes, key genes in ethylene biosynthesis, had low expression levels and similar expression patterns in cultivated and wild watermelon, as well as very low levels of released ethylene in watermelon fruit, further functional studies are required to investigate the exact roles of these ethylene pathway genes in non-climacteric watermelon fruit ripening.

## Conclusions

Watermelon is an important fruit crop and becomes a model system for studies of non-climacteric fruits, while the molecular mechanisms of its fruit development and ripening remain largely unknown. Genome-wide comparative gene expression analysis between the cultivated and wild watermelon would provide a better understanding of the genetic bases contributing to the differences of their fruit development. Using the high-throughput RNA-Seq technology, we have generated approximately 321.8 million high quality reads from key stages of fruit development in both cultivated and wild watermelon. This dataset provided a much more elaborate transcriptional status during watermelon fruit development, and for the first time, provided gene expression profiles during fruit development of a wild watermelon. Analysis of gene expression profiles indicated that many of the processes associated with watermelon fruit quality were regulated at the transcriptional level. Comparative RNA-Seq analysis between cultivated and wild watermelon identified a number of key genes potentially involved in determining watermelon fruit qualities including sugar metabolism and transport, carotenoid biosynthesis, fiber and pectin degeneration. Some of these genes are the direct target of the regulators of watermelon fruit qualities thus our analysis provides an infrastructure that could lead to the identification of the causal genes. Regulatory genes, such as those in the ethylene biosynthesis and signaling pathway with highly ripening-associated expression patterns were also identified. Expression dynamics of these genes indicated that ethylene might be involved in regulating watermelon fruit quality although watermelon is classified as a non-climacteric fruit. Our comparative transcriptome analysis provided genome-wide novel insights into the molecular mechanisms of fruit development and ripening and the regulatory mechanisms of several important fruit quality traits of watermelon such as sugar accumulation, flesh color and flesh texture.

## Supporting Information

S1 Fig Amino acid sequence alignment of watermelon α-galactosidases and the melon α-galactosidase *CmAGA2*. (TIF)

S1 Table Primer pairs used for qRT-PCR analysis. (XLSX)

S2 Table Mesocarp sugar content and firmness of 97103 and PI296341-FR. (XLSX)

S3 Table Genes differentially expressed during watermelon fruit development. (XLSX)

S4 Table Validation of RNA-Seq results by qRT-PCR. (XLSX)

S5 Table GO terms enriched in genes differentially expressed during watermelon fruit development. (XLSX)

S6 Table Significantly changed pathways represented by the genes differentially expressed during watermelon fruit development. (XLSX)

S7 Table Sugar metabolic genes and transporters that were differentially expressed during watermelon fruit development. (XLSX)

S8 Table Carotenoid content in fruit flesh during watermelon fruit development. (XLSX)

S9 Table Carotenoid metabolic genes that were differentially expressed during watermelon fruit development. (XLSX)

S10 Table Flesh texture related genes that were differentially expressed during watermelon fruit development. (XLSX)

S11 Table Ethylene biosynthesis and signal transduction genes that were differentially expressed during watermelon fruit development. (XLSX)

## References

[1] Liu J, Guo S, He H, Zhang H, Gong G, Ren Y, et al Dynamic characteristics of sugar accumulation and related enzyme activities in sweet and non-sweet watermelon fruits. Acta Physiol Plant. 2013;35: 3213–3222. 10.1007/s11738-013-1356-0

[2] FAO Statistics. Available: http://faostat.fao.org.

[3] Wechter WP, Levi A, Harris KR, Davis AR, Fei Z, Katzir N, et al Gene expression in developing watermelon fruit. BMC Genomics. 2008;9: 275 10.1186/1471-2164-9-275 18534026 PMC2440768

[4] Guo S, Liu J, Zheng Y, Huang M, Zhang H, Gong G, et al Characterization of transcriptome dynamics during watermelon fruit development: sequencing, assembly, annotation and gene expression profiles. BMC Genomics. 2011;12: 454 10.1186/1471-2164-12-454 21936920 PMC3197533

[5] Yativ M, Harary I, Wolf S. Sucrose accumulation in watermelon fruits: genetic variation and biochemical analysis. J Plant Physiol. 2010;167: 589–596. 10.1016/j.jplph.2009.11.009 20036442

[6] Guo S, Zhang J, Sun H, Salse J, Lucas WJ, Zhang H, et al The draft genome of watermelon (Citrullus lanatus) and resequencing of 20 diverse accessions. Nat Genet. 2013;45: 51–58. 10.1038/ng.2470 23179023

[7] Zhong S, Joung JG, Zheng Y, Chen YR, Liu B, Shao Y, et al High-throughput illumina strand-specific RNA sequencing library preparation. Cold Spring Harb Protoc. 2011;2011: 940–949. 10.1101/pdb.prot5652 21807852

[8] Bolger AM, Lohse M, Usadel B. Trimmomatic: a flexible trimmer for Illumina sequence data. Bioinformatics. 2014;30: 2114–2120. 10.1093/bioinformatics/btu170 24695404 PMC4103590

[9] Quast C, Pruesse E, Yilmaz P, Gerken J, Schweer T, Yarza P, et al The SILVA ribosomal RNA gene database project: improved data processing and web-based tools. Nucleic Acids Res. 2013;41: D590–D596. 10.1093/nar/gks1219 23193283 PMC3531112

[10] Langmead B, Trapnell C, Pop M, Salzberg S. Ultrafast and memory-efficient alignment of short DNA sequences to the human genome. Genome Biol. 2009;10: R25 10.1186/gb-2009-10-3-r25 19261174 PMC2690996

[11] Trapnell C, Pachter L, Salzberg SL. TopHat: discovering splice junctions with RNA-Seq. Bioinformatics. 2009;25: 1105–1111. 10.1093/bioinformatics/btp120 19289445 PMC2672628

[12] Anders S, Huber W. Differential expression analysis for sequence count data. Genome Biol. 2010;11: R106 10.1186/gb-2010-11-10-r106 20979621 PMC3218662

[13] Smyth GK. Linear models and empirical bayes methods for assessing differential expression in microarray experiments. Stat Appl Genet Mol Biol. 2004;3: Article3 10.2202/1544-6115.1027 16646809

[14] Y. B, Y. H. Controlling the False Discovery Rate: A Practical and Powerful Approach to Multiple Testing. J R Stat Soc Series B Stat Methodol. 1995;57: 289–300. 10.2307/2346101

[15] Boyle EI, Weng S, Gollub J, Jin H, Botstein D, Cherry JM, et al GO::TermFinder—open source software for accessing Gene Ontology information and finding significantly enriched Gene Ontology terms associated with a list of genes. Bioinformatics. 2004;20: 3710–3715. 10.1093/bioinformatics/bth456 15297299 PMC3037731

[16] Joung J-G, Corbett AM, Fellman SM, Tieman DM, Klee HJ, Giovannoni JJ, et al Plant MetGenMAP: An Integrative Analysis System for Plant Systems Biology. Plant Physiol. 2009;151: 1758–1768. 10.1104/pp.109.145169 19819981 PMC2786002

[17] Schmittgen TD, Livak KJ. Analyzing real-time PCR data by the comparative CT method. Nat Protoc. 2008;3: 1101–1108. 10.1038/nprot.2008.73 18546601

[18] Fraser PD, Truesdale MR, Bird CR, Schuch W, Bramley PM. Carotenoid Biosynthesis during Tomato Fruit Development (Evidence for Tissue-Specific Gene Expression). Plant Physiol. 1994;105: 405–413. 10.1104/pp.105.1.405 12232210 PMC159369

[19] Grassi S, Piro G, Lee JM, Zheng Y, Fei Z, Dalessandro G, et al Comparative genomics reveals candidate carotenoid pathway regulators of ripening watermelon fruit. BMC Genomics. 2013;14: 781 10.1186/1471-2164-14-781 24219562 PMC3840736

[20] Wang Z, Gerstein M, Snyder M. RNA-Seq: a revolutionary tool for transcriptomics. Nat Rev Genet. 2009;10: 57–63. 10.1038/nrg2484 19015660 PMC2949280

[21] Koenig D, Jimenez-Gomez JM, Kimura S, Fulop D, Chitwood DH, Headland LR, et al Comparative transcriptomics reveals patterns of selection in domesticated and wild tomato. Proc Natl Acad Sci U S A. 2013;110: E2655–2662. 10.1073/pnas.1309606110 23803858 PMC3710864

[22] Lee JM, Joung JG, McQuinn R, Chung MY, Fei Z, Tieman D, et al Combined transcriptome, genetic diversity and metabolite profiling in tomato fruit reveals that the ethylene response factor SlERF6 plays an important role in ripening and carotenoid accumulation. Plant J. 2012;70: 191–204. 10.1111/j.1365-313X.2011.04863.x 22111515

[23] Castro-Rodriguez V, Garcia-Gutierrez A, Canales J, Avila C, Kirby E, Canovas F. The glutamine synthetase gene family in Populus. BMC Plant Biol. 2011;11: 119 10.1186/1471-2229-11-119 21867507 PMC3224142

[24] Avila C, Suárez MF, Gómez-Maldonado J, Cánovas FM. Spatial and temporal expression of two cytosolic glutamine synthetase genes in Scots pine: functional implications on nitrogen metabolism during early stages of conifer development. Plant J. 2001;25: 93–102. 10.1111/j.1365-313X.2001.00938.x 11169185

[25] Mitchell DE, Gadus MV, Madore MA. Patterns of Assimilate Production and Translocation in Muskmelon (Cucumis melo L.): I. Diurnal Patterns. Plant Physiol. 1992;99: 959–965. 10.1104/pp.99.3.959 16669025 PMC1080570

[26] Schaffer AA, Pharr DM, Madore MA. Cucurbits In: Zamski E, Schaffer AA, editors. Photoassimilate Distribution in Plants and Crops: Source-Sink Relationships. New York Marcel Dekker; 1996 pp. 729–757.

[27] Hubbard NL, Huber SC, Pharr DM. Sucrose Phosphate Synthase and Acid Invertase as Determinants of Sucrose Concentration in Developing Muskmelon (Cucumis melo L.) Fruits. Plant Physiol. 1989;91: 1527–1534. 10.1104/pp.91.4.1527 16667212 PMC1062217

[28] Gao Z, Schaffer AA. A novel alkaline alpha-galactosidase from melon fruit with a substrate preference for raffinose. Plant Physiol. 1999;119: 979–988. 10.1104/pp.119.3.979 10069835 PMC32111

[29] Carmi N, Zhang G, Petreikov M, Gao Z, Eyal Y, Granot D, et al Cloning and functional expression of alkaline alpha-galactosidase from melon fruit: similarity to plant SIP proteins uncovers a novel family of plant glycosyl hydrolases. Plant J. 2003;33: 97–106. 10.1046/j.1365-313X.2003.01609.x 12943544

[30] Godt DE, Roitsch T. Regulation and tissue-specific distribution of mRNAs for three extracellular invertase isoenzymes of tomato suggests an important function in establishing and maintaining sink metabolism. Plant Physiol. 1997;115: 273–282. 10.1104/pp.115.1.273 9306701 PMC158483

[31] Tang GQ, Lüscher M, Sturm A. Antisense Repression of Vacuolar and Cell Wall Invertase in Transgenic Carrot Alters Early Plant Development and Sucrose Partitioning. Plant Cell. 1999;11: 177–189. 10.1105/tpc.11.2.177 9927637 PMC144160

[32] Dai N, Petreikov M, Portnoy V, Katzir N, Pharr DM, Schaffer AA. Cloning and expression analysis of a UDP-galactose/glucose pyrophosphorylase from melon fruit provides evidence for the major metabolic pathway of galactose metabolism in raffinose oligosaccharide metabolizing plants. Plant Physiol. 2006;142: 294–304. 10.1104/pp.106.083634 16829585 PMC1557607

[33] Ren Y, McGregor C, Zhang Y, Gong G, Zhang H, Guo S, et al An integrated genetic map based on four mapping populations and quantitative trait loci associated with economically important traits in watermelon (Citrullus lanatus). BMC Plant Biol. 2014;14: 33 10.1186/1471-2229-14-33 24443961 PMC3898567

[34] Rao AV, Rao LG. Carotenoids and human health. Pharmacol Res. 2007;55: 207–216. 10.1016/j.phrs.2007.01.012 17349800

[35] Holden JM, Eldridge AL, Beecher GR, Marilyn Buzzard I, Bhagwat S, Davis CS, et al Carotenoid Content of U.S. Foods: An Update of the Database. J Food Compost Anal. 1999;12: 169–196. 10.1006/jfca.1999.0827

[36] Schwartz SH, Tan BC, Gage DA, Zeevaart JAD, McCarty DR. Specific Oxidative Cleavage of Carotenoids by VP14 of Maize. Science. 1997;276: 1872–1874. 10.1126/science.276.5320.1872 9188535

[37] Schwartz SH, Qin X, Zeevaart JA. Elucidation of the indirect pathway of abscisic acid biosynthesis by mutants, genes, and enzymes. Plant Physiol. 2003;131: 1591–1601. 10.1104/pp.102.017921 12692318 PMC1540303

[38] Li Q, Li P, Sun L, Wang Y, Ji K, Sun Y, et al Expression analysis of beta-glucosidase genes that regulate abscisic acid homeostasis during watermelon (Citrullus lanatus) development and under stress conditions. J Plant Physiol. 2012;169: 78–85. 10.1016/j.jplph.2011.08.005 21940067

[39] Winterhalter P, Rouseff R. Carotenoid-derived aroma compounds: an introduction In: Winterhalter P, Rouseff, editors. Carotenoid-derived aroma compounds. Washington, DC, American Chemical Society; 2002 pp. 1–19.

[40] Brownleader MD, Jackson P, Mobasheri A, Pantelides AT, Sumar S, Trevan M, et al Molecular Aspects of Cell Wall Modifications during Fruit Ripening. Crit Rev Food Sci Nutr. 1999;39: 149–164. 10.1080/10408399908500494 10198752

[41] Bartley IM, Knee M. The chemistry of textural changes in fruit during storage. Food Chem. 1982;9: 47–58. 10.1016/0308-8146(82)90068-1

[42] Brummell DA, Dal Cin V, Crisosto CH, Labavitch JM. Cell wall metabolism during maturation, ripening and senescence of peach fruit. J Exp Bot. 2004;55: 2029–2039. 10.1093/jxb/erh227 15286150

[43] Brummell DA. Cell wall disassembly in ripening fruit. Funct Plant Biol. 2006;33: 103–119. 10.1071/FP05234 32689218

[44] Brummell DA, Harpster MH. Cell wall metabolism in fruit softening and quality and its manipulation in transgenic plants. Plant Mol Biol. 2001;47: 311–340. 10.1023/A:1010656104304 11554479

[45] Liu J, He H, Guo S, Zhang H, Ren Y, Gong G, et al Physiological and biochemical mechanism for watermelon fruit ripening and softening. Journal of Fruit Science. 2013;30: 813–818. 10.13925/j.cnki.gsxb.2013.05.015 23695311

[46] Soltani B, Ehlting J, Hamberger B, Douglas C. Multiple cis-regulatory elements regulate distinct and complex patterns of developmental and wound-induced expression of Arabidopsis thaliana 4CL gene family members. Planta. 2006;224: 1226–1238. 10.1007/s00425-006-0296-y 16738863

[47] Saxena IM, Brown RM. Cellulose Biosynthesis: Current Views and Evolving Concepts. Ann Bot. 2005;96: 9–21. 10.1093/aob/mci155 15894551 PMC4246814

[48] Vicente AR, Ortugno C, Powell ALT, Greve LC, Labavitch JM. Temporal Sequence of Cell Wall Disassembly Events in Developing Fruits. 1. Analysis of Raspberry (Rubus idaeus). J Agric Food Chem. 2007;55: 4119–4124. 10.1021/jf063547r 17428067

[49] Vicente AR, Powell A, Greve LC, Labavitch JM. Cell wall disassembly events in boysenberry (Rubus idaeus L. × Rubus ursinus Cham. &amp; Schldl.) fruit development. Funct Plant Biol. 2007;34: 614–623. 10.1071/FP07002 32689389

[50] Jolie RP, Duvetter T, Van Loey AM, Hendrickx ME. Pectin methylesterase and its proteinaceous inhibitor: a review. Carbohydr Res. 2010;345: 2583–2595. 10.1016/j.carres.2010.10.002 21047623

[51] Pinzon-Latorre D, Deyholos M. Characterization and transcript profiling of the pectin methylesterase (PME) and pectin methylesterase inhibitor (PMEI) gene families in flax (Linum usitatissimum). BMC Genomics. 2013;14: 742 10.1186/1471-2164-14-742 24168262 PMC4008260

[52] Di Matteo A, Giovane A, Raiola A, Camardella L, Bonivento D, De Lorenzo G, et al Structural Basis for the Interaction between Pectin Methylesterase and a Specific Inhibitor Protein. Plant Cell. 2005;17: 849–858. 10.1105/tpc.104.028886 15722470 PMC1069703

[53] Yashoda HM, Prabha TN, Tharanathan RN. Mango ripening–Role of carbohydrases in tissue softening. Food Chem. 2007;102: 691–698. 10.1016/j.foodchem.2006.06.001

[54] Meli VS, Ghosh S, Prabha TN, Chakraborty N, Chakraborty S, Datta A. Enhancement of fruit shelf life by suppressing N-glycan processing enzymes. Proc Natl Acad Sci U S A. 2010;107: 2413–2418. 10.1073/pnas.0909329107 20133661 PMC2823905

[55] Lelièvre J-M, Latchè A, Jones B, Bouzayen M, Pech J-C. Ethylene and fruit ripening. Physiol Plant. 1997;101: 727–739. 10.1111/j.1399-3054.1997.tb01057.x

[56] Iannetta PPM, Laarhoven L-J, Medina-Escobar N, James EK, McManus MT, Davies HV, et al Ethylene and carbon dioxide production by developing strawberries show a correlative pattern that is indicative of ripening climacteric fruit. Physiol Plant. 2006;127: 247–259. 10.1111/j.1399-3054.2006.00656.x

[57] Trainotti L, Pavanello A, Casadoro G. Different ethylene receptors show an increased expression during the ripening of strawberries: does such an increment imply a role for ethylene in the ripening of these non-climacteric fruits? J Exp Bot. 2005;56: 2037–2046. 10.1093/jxb/eri202 15955790

[58] Katz E, Lagunes P, Riov J, Weiss D, Goldschmidt E. Molecular and physiological evidence suggests the existence of a system II-like pathway of ethylene production in non-climacteric Citrus fruit. Planta. 2004;219: 243–252. 10.1007/s00425-004-1228-3 15014996

[59] Elkashif ME, Huber DJ. Enzymic hydrolysis of placental cell wall pectins and cell separation in watermelon (Citrullus lanatus) fruits exposed to ethylene. Physiol Plant. 1988;73: 432–439. 10.1111/j.1399-3054.1988.tb00622.x

[60] Yasar K, J HD. Cell wall-degrading enzymes and pectin solubility and depolymerization in immature and ripe watermelon (Citrullus lanatus) fruit in response to exogenous ethylene. Physiol Plant. 2002;116: 398–405. 10.1034/j.1399-3054.2002.1160316.x

[61] Chung MY, Vrebalov J, Alba R, Lee J, McQuinn R, Chung JD, et al A tomato (Solanum lycopersicum) APETALA2/ERF gene, SlAP2a, is a negative regulator of fruit ripening. Plant J. 2010;64: 936–947. 10.1111/j.1365-313X.2010.04384.x 21143675

[62] Cara B, Giovannoni JJ. Molecular biology of ethylene during tomato fruit development and maturation. Plant Sci. 2008;175: 106–113. 10.1016/j.plantsci.2008.03.021

[63] Xiao YY, Chen JY, Kuang JF, Shan W, Xie H, Jiang YM, et al Banana ethylene response factors are involved in fruit ripening through their interactions with ethylene biosynthesis genes. J Exp Bot. 2013;64: 2499–2510. 10.1093/jxb/ert108 23599278 PMC3654433

