# Supplementary figures and images for: Comparative Transcriptome Analysis of Cultivated and Wild Watermelon during Fruit Development
Source: PLoS One. 2015 Jun 16;10(6):e0130267. doi: 10.1371/journal.pone.0130267 (PMC4469606; doi:10.1371/journal.pone.0130267)

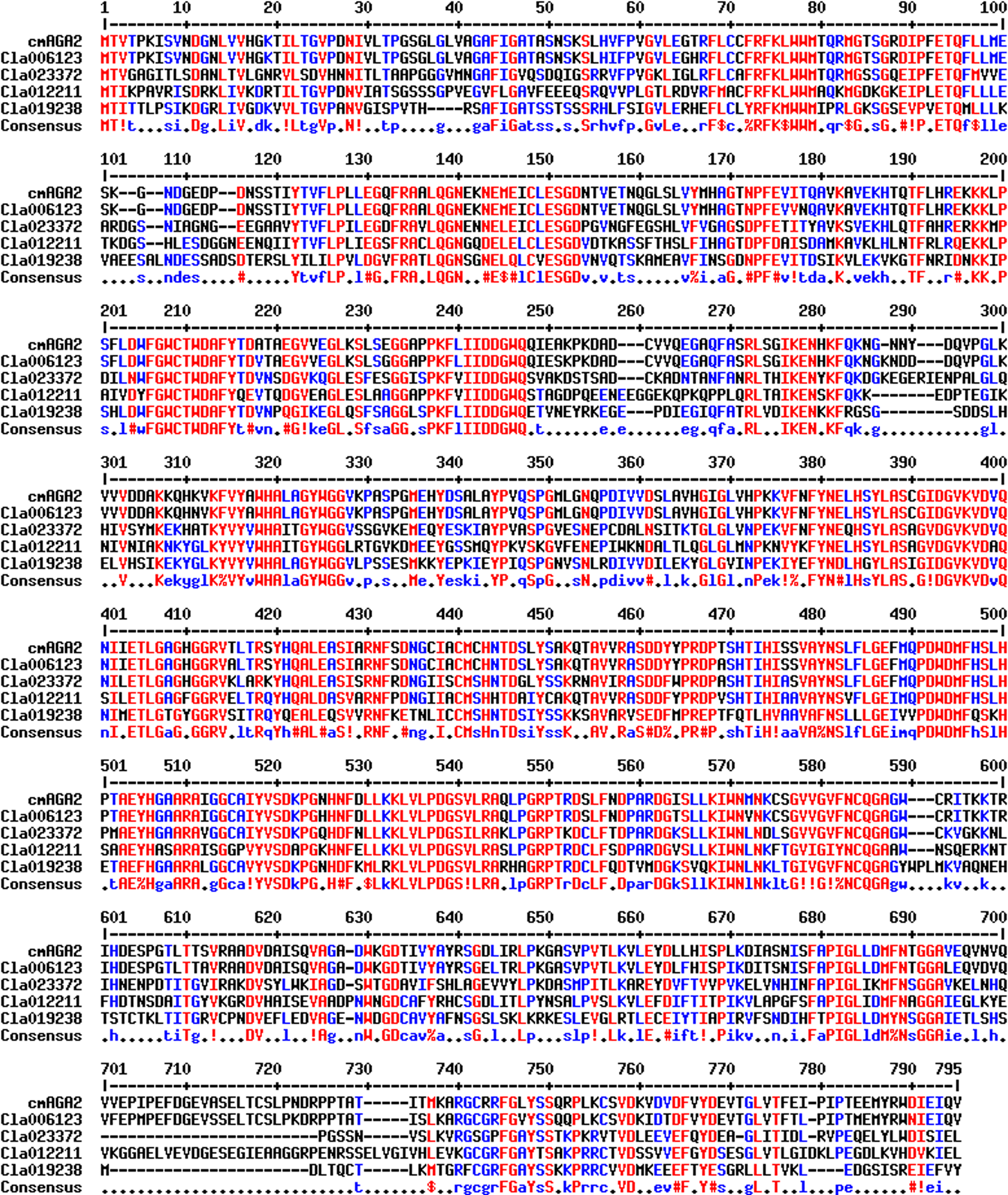

Supplement: S1 Fig — (TIF) [file pone.0130267.s001.tif]
